# The Physician’s Leisure; a Plea for the Study of Archæology*This address was written about two years ago, at the request of the author’s friends, the pupils of a medical school, with a view to its being read at their physical society. This intention was not carried out; and it now appears that the opening of a session is a proper occasion for bringing the subject forward.

**Published:** 1881-05

**Authors:** 


### Selections.

THE PHYSICIAN'S LEISURE; A PLEA FOR THE STUDY OF ARCHÆOLOGY.* By DR. CHEVERS.

*Gentlemen:*—When an old physician is asked by a young one, or by a student of medicine, to advise him with regard to his course in life, as you have requested me, one of the soundest, yet perhaps not the most courtly or most original, pieces of good counsel that he can offer is—"*Mind your business!*" Still, this monition would be very imperfect, nay, almost useless, if allowed to stand alone without its proper and natural adjunct, "*Allow yourself a small, but fair, complement of leisure, and occupy that leisure to the best possible advantage.*"

In a long experience, I have known several medical men who had no occupation except the practice of their profession. Most of these were deservedly successful workers; and so far, you will say, they did wisely and well. Still, I noticed that those intervals of business, which must and ought to occur rather frequently in the life of every active man, appeared to be scarcely employed with advantage by them. They could not read, they were not artists or chess-players. They smoked or chatted, or played billiards—which no gentleman in India, or, let us trust, elsewhere, ever does in a public room. And thus they obtained both mental and physical rest; but that rest was certainly not of a high or intellectual order. As a means of recuperating the tired mind it could hardly be regarded as more valid than sleep. Had

* This address was written about two years ago, at the request of the author's friends, the pupils of a medical school, with a view to its being read at their physical society. This intention was not carried out; and it now appears that the opening of a session is a proper occasion for bringing the subject forward.

they taken long walks or rides, I should have thought that they occupied their leisure better. Still, it has always appeared to me that, except it be taken *with an object*, a ride or walk is a simply tiresome proceeding. The medical man who delights in such exercise ought to be a botanist, an entomologist, a geologist, or an antiquary; or he ought, at least, to follow the hounds, or the trout, or the partridges.

Again, the physician who is most devoted to his profession must occasionally have other and longer intervals of rest forced upon him. He falls ill; or, having made his fortune, he retires. Where, under these circumstances, is his intellectual life? What charming prospect of well-earned and long-desired healthy mental and physical recreation lies before him? His mind has been trained to work only in a single deep but narrow groove, and now the way of its advance is actually barred. You will say he may, even in permanent retirement, continue to be a student in his profession; there is always plenty to learn in medicine as the science makes progress. No! That track is closed against him. The more a practitioner has loved the cultivation of his profession, for its own sake, in active life, the less he cares to study it in retirement unless he be himself a writer. To watch passively the stately onward march of that science of which he has ceased to be a leader and a master, and to sit idly watching the daily race in which he, yesterday, an athlete, is now paralyzed as far as active competition and valid advance are concerned, produces in his mind a sense of natural, though confessedly selfish, pain. He says, "I am left behind; I am forgotten!" or, in a nobler frame of mind, he sighs regretfully, and uses the words which a poet has given to Joan of Arc in prison—

"I am God's broken sword!"

"It is no longer granted to me to administer the noble and saving power with which I was entrusted by my Maker; the great object of my life has been snatched from my grasp; I am blind, or a cripple, or an aged man, and now my existence affords me no prospect of rational pleasure!" It may certainly be argued, the man whom you pictured just now is not, in the first place, a very highly cultured, intellectual being, and you imagine for him regrets which he is scarcely capable of entertaining;—dominoes remain perfectly open to him, or he will probably be well satisfied to decline into "an old age of cards." Still we must remember that the majority of ourselves are only average people; and I think it incumbent upon each of us to consider how that rest and leisure which are so needful, and so exquisitely welcome and delightful to the majority of working men, can be prevented from becoming to us a mere shunting into some Sleepy Hollow, in the fruition of which the very hybernating animals are our superiors.

Some of you may then inquire of me—How am I to elect the best means of employing my leisure? To this I must reply—Nature will often teach you this. From your earliest youth you have possessed an ear for music, or a tendency to draw upon your slate, or a love for healthy field sports and the best pleasures of a country life. Thus constituted, you are fortunate men, and there is nothing in the profession of medicine that refuses to allow you to devote much of your leisure to music, drawing, hunting, shooting, cricketing, or boating. Or, happily, you may start in life with natural gifts which are, to the physician (drawing being excepted), far more appropriate than these. You may be endowed with a mechanical gift, or you may feel a keen interest in all that relates to the lower animals, or you may so love reading that scarcely any book fails to interest you. It was by the cultivation of such natural instinctive tendencies as these that William Fergusson, John Hunter, and James Copland did so much towards rendering our science illustrious. If he had the making of his own mind, every medical man ought to become either a zoologist, a geologist, a botanist, a chemist, a draughtsman, or a scientific mechanician; and should, in the pursuit of one of these collateral sciences, find ample occupation for his leisure. But, certainly most fortunately, we are not all self-made; a natural, inborn taste, however excellent, cannot be forced upon us; and not a few of us must confess, with regret, that there are, among the beautiful sciences collateral to medicine, more than one the full acquisition of which is practically impossible to us. Still, we must all, if we wish to pass the dawn and the evening of our active lives pleasantly as well as successfully, look around us for some soft and smooth pillow upon which the aching brow and the overtasked mind can seek repose and recover working energy.

A very sad fate, not at all well understood by the public, and hardly enough recognized by the profession, not unfrequently falls upon a man of the grandest intellect. In his case old age advances, and with it reputation suddenly rises to its highest point, and work comes in with almost overwhelming pressure. In that once admirably organized mind and body senile changes have set in, decay has commenced, the heart is somewhat weakened, the cerebral arteries are becoming narrow and rigid: but the artist's picture must be finished for the exhibition of the 1st of May; the physician's visiting-list is so overwhelmingly full of appointments that he almost sobs as he shuts his diary, knowing that, immediately he declares himself unequal to work, practice will leave him; the writer of brilliant leaders is irrevocably pledged to insert so many columns, and not one less, on the most intricate question of the day, in to-morrow's journal; the judge finds himself within twenty hours of the task of summing up in his court the evidence which has occupied the attention of a jury and of all Europe for many weeks. All these are met by one Satanic prescription. The Tempter whispers, "Drink port wine, my learned or artistic friend, as did more than one great painter whose history is too recent and too sad to need further mention, and as Lord Stowell did; try gin-and-water, used-up essayist, as Lord Byron did, and find in it immediate inspiration!" And, fatally, so it is. The trembling hand is steadied, and throws upon the canvas a glow such as Nature rarely sheds over sky and sea and wold; for three hours to-morrow the aged voice will ring clearly down from the seat of judgment in broadly decisive arguments which will stand in the law reports, as irrevocable judgments, for a century to come; and thus, for a few more months or years, the sad, evil, and shameful succession, the *facile descensus*, will be—stimulation, effort, collapse, and then stimulation again, until apoplexy, delirium, or suicide closes the miserable and most unfair struggle. And then the world will say—"He was a man of genius; but, latterly at least, a wretched sot!" We may trust that He who made and endowed him, who raised him into a position to which very few but a Gull, a Cockburn, or a Beaconsfield are fully equal, and who knows how fallible he was and how sorely tried, will award him a juster verdict. What now ought either of these unhappy men to have done to escape the threatened fate? Should he not have retired from business before the beginning of the end set in, occupying, as James Blundell and William Macready did, and as James Annesley and Edward Waring and several other Indian physicians have done, a long retirement in literary leisure? Failing the means to do this, should he not, like Sir Henry Holland, be content with moderate success and wealth, and devote a portion of each year to foreign travel, the best of all rests and tonics to a worn brain and a feeble digestion? Or, with less time to spare, ought he not to seek a more vigorous life (or, it may be, find death, as they found it) in the saddle, as Samuel Wilberforce did, or as John Liston did, in his yacht on the Thames? Or, allowing himself still fewer spare moments, might he not follow the example of Richard Bright or Francis Sibson, and spend some of his evenings in delightful contemplation of his folios of early German engravings or his cabinets of Wedgwood ware?

These latter allusions lead me to the proposition that he who rest his mind by judiciously employing his leisure as a collector often finds that he can work more steadily and better and farther on in life than many of his neighbors do. The "collector," of sound principles and well-balanced mind, leaves his work as gaily and as hopefully as the boy leaves his school-house, and returns to it again bright, active, and refreshed. In pursuing his collection, there is no part of the town or of the country to which he does not penetrate, full of thought and of pleasant inquiry and interest. Whether he is in search of a rare postage stamp, or a dog's-eared little Elzevir, or a plant which has taken up its habitat in some almost inaccessible spot; or whether, like Joe Grimaldi, he starts after the pantomime is over and walks nineteen miles to Dartford that he may be in time to catch a Camberwell Beauty in the early morning, his mind is never in want of a desirable and exhilarating object of pursuit, while his body is refreshed by the most invigorating exercise.

I can scarcely imagine a drier-mannered and more reticent little old philosopher, or one who, in his public life, gave one the idea of being less capable of amusing himself, than Richard Bright, the expositor of Morbus Brightii, was. I was lately surprised to learn, from a leading print-seller, that, often as late as twelve o'clock at night, Dr. Bright would knock him up and say, "Now, Mr. Faussett, let me see those engravings. I have not had a minute to spare all day long!" He was a diligent and judicious print-collector.

But the maxim that the active collector is generally a bright and indefatigable worker, is worse than useless without the correction that *he must not allow his passion for collecting to encroach either upon his business or his means.* As a general rule, a not very rich but conscientious collector is, in all his affairs, collecting, classifying, and assorting, in his business and in his household, a man of order and method; and, if it be only to find the means of collecting, he is usually industrious, economical, and moderate in all things. But collecting may become an engrossing, overpowering passion, and passion is only one remove in kinship from vice. Some years ago, an English nobleman resolved that he possessed taste, and became a collector. Now, when a well-to-do Duke determines that his collection of pictures, or of statuary, or of gold medals, or of antique gems, or his conservatories, shall be the finest in the world, he encounters a very stiff but not quite impracticable task; but, unhappily, this magnate attempted all these collections at once, and, very naturally, discovered, rather early in his career, that his magnificent revenues were seriously compromised.

The collection of medal portraits in the library of our College of Surgeons was made by a banker, Henry Fauntleroy (who, after he was hanged, was unkindly called Corduroy). But, although a man of taste, this individual was self-indulgent and cruel as a Sybarite. His passion for collecting and other less happy proclivities brought his career to the consummation which I have indicated; but, as Sir Joseph Fayer has shown that every venomous Indian serpent has a harmless snake which closely resembles it, so every virtue has its corresponding vice.

These remarks will prepare you to receive the proposition that a love for Archæology is calculated to afford a delightful solace to the hard-working medical man. Several of you may be so considerate as to take up this proposition for the moment, and to inquire—How am I to become an antiquary?

I will at once put your question to a crucial test. In amusing yourself in your garden, would you rather find a spade-guinea of last century or a lar of Mars? Or rather, in estimating one object against the other, would you value the coin because it is an object of a certain intrinsic value and a pretty appendage to the watch-chain, and despise the other because it is a not very artistic little image of ugly bronze, not intrinsically worth its weight in copper, with the erugo thick upon it? If so, you have not a spice of true antiquarian enthusiasm, and I can only beg you to endure me as I proceed, but never to attempt to learn to become that for which the natural bent of your mind has not fitted you. I conscientiously believe that, whenever a medical man can take kindly to the study of antiquities,—it need not, to be useful to the individual, go beyond mere dilettantism,—it will not only afford him life-long pleasure, but will, properly directed, validly aid him in the study of his profession. It is a mere platitude to declare that a physician who knows nothing of the history of medicine, is as ignorant and narrow-minded a professor as is the statesman who is unacquainted with the political history of his country. The history of every branch of the profession which you and I have happily embraced is full of interesting and valuable teaching; for it must be remembered that our predecessors, although imperfectly grounded in physiology and pathology, and by no means free from credulity and superstition, were often acute, cultivated, observant, and deeply reflecting men, who frequently obtained glimpses of great truths which, having been imperfectly perceived, lapsed into oblivion for centuries. Take a few points, out of hundreds, in several branches of our science. Thus, in Surgery, English surgeons, a hundred years ago, had no specula; the Pompeian surgeons had. Peter Lowe, surgeon to Henry the Fourth of France, taught that the proper treatment in strangulated and incarcerated hernia is puncture; which has, of late, been used with some advantage in cases of intestinal obstruction.

[TO BE CONCLUDED.]

